# Removal of Cr(VI) from Wastewater Using Graphene Oxide Chitosan Microspheres Modified with α–FeO(OH)

**DOI:** 10.3390/ma15144909

**Published:** 2022-07-14

**Authors:** Yunquan Liu, Huimei Shan, Chunya Zeng, Hongbin Zhan, Yanyue Pang

**Affiliations:** 1 Guangxi Key Laboratory of Environmental Pollution Control Theory and Technology, Guilin University of Technology, Guilin 541004, China; buddychampion@163.com (Y.L.); 15078381513@163.com (C.Z.); pangyanyue080512@163.com (Y.P.); 2 Collaborative Innovation Center of Water Pollution Control and Water Security in Karst Area, Guilin University of Technology, Guilin 541004, China; 3 College of Environmental Science and Engineering, Guilin University of Technology, Guilin 541004, China; 4 Department of Geology and Geophysics, Texas A&M University, College Station, TX 77843, USA

**Keywords:** hydroxyl iron oxide, graphene oxide, chitosan, Cr(VI) adsorption

## Abstract

Graphene oxide and chitosan microspheres modified with α–FeO(OH) (α–FeO(OH)/GOCS) are prepared and utilized to investigate the performance and mechanism for Cr(VI) removal from aqueous solutions and the possibility of Fe secondary pollution. Batch experiments were carried out to identify the effects of pH, mass, and volume ratio (*m*/*v*), coexisting ions, time (*t*), temperature (*T*), and Cr(VI) initial concentration (*C*_0_) on Cr(VI) removal, and to evaluate adsorption kinetics, equilibrium isotherm, and thermodynamics, as well as the possibility of Fe secondary pollution. The results showed that Cr(VI) adsorption increased with *C*_0_, *t*, and *T* but decreased with increasing pH and *m*/*v*. Coexisting ions inhibited Cr(VI) adsorption, and this inhibition increased with increasing concentration. The influence degrees of anions and cations on the Cr(VI) adsorption in descending order were SO_4_^2−^ > PO_4_^2−^ > NO_3_^−^ > Cl^−^ and Ca^2+^ > Mg^2+^ > Mn^2+^, respectively. The equilibrium adsorption capacity of Cr(VI) was the highest at 24.16 mg/g, and the removal rate was 97.69% under pH = 3, *m*/*v* = 1.0 g/L, *T* = 298.15 K, and *C*_0_ = 25 mg/L. Cr(VI) adsorption was well fitted to a pseudo-second-order kinetic model and was spontaneous and endothermic. The best fit of Cr(VI) adsorption with the Langmuir and Sips models indicated that it was a monolayer and heterogeneous adsorption. The fitted maximum adsorption capacity was 63.19 mg/g using the Sips model under 308.15 K. Cr(VI) removal mainly included electrostatic attraction between Cr(VI) oxyanions with surface Fe–OH_2_^+^, and the adsorbed Cr(VI) was partially reduced to Cr(III) and then precipitated on the surface. In addition, there was no Fe secondary pollution during Cr(VI) adsorption.

## 1. Introduction

Heavy metal pollution in wastewater is becoming severe with the rapid development of industry and has become a widespread concern worldwide. Heavy metals mostly refer to elements with significant biological toxicity, such as mercury, cadmium, lead, chromium, and metal-like arsenic [1]. Chromium pollution is an increasingly prominent problem and widely occurs in industrial wastewater, involving metal processing, leather tanning, electroplating, and pigment manufacturing [2]. The two main oxidation states of chromium in wastewater are hexavalent chromium (Cr(VI)) and trivalent chromium (Cr(III)) [3], and Cr(VI) is more dangerous to human health because it has stronger carcinogenicity, teratogenicity, and mutagenicity [4]. The species of Cr(VI) in wastewater include CrO_4_^2−^, Cr_2_O_7_^2−^, HCrO_4_^−^, and H_2_CrO_4_ [5]. The World Health Organization (WHO) has suggested that the limitation of chromium concentration in drinking water is 0.05 mg/L [6]. Therefore, it is necessary to develop effective methods to remove Cr(VI) from wastewater.

Recently, many methods have been applied to remove Cr(VI) from wastewater, including chemical precipitation, electrolysis, ion exchange, membrane filtration, adsorption, and photocatalytic reduction [7,8,9,10]. The adsorption method is regarded as one of the most popular methods due to its advantages of simple operation, low energy consumption, and environmental friendliness [11]. Many traditional adsorption materials have been widely used for Cr(VI) removal, including activated carbon [12], zeolite [13], clay minerals [14] and ion exchange resins [15], etc. Although these materials are highly efficient in removing Cr(VI), they are difficult to recycle and may cause secondary pollution problems [16]. Therefore, it is necessary to design and develop new types of adsorbents with better stability and to avoid secondary pollution.

Graphene oxide (GO) is a new nanomaterial that has a large surface area (theoretically ~2600 m^2^/g) [17] and is rich in oxygen functional groups (such as −OH, −C=O, and −COOH) that can bind well with heavy metal ions [18]. In addition, it has been confirmed that the modification of GO surface functional groups has a positive effect on Cr(VI) removal [19]. However, GO also has some limitations, such as poor separation and easy self-aggregation [20]. Therefore, it is necessary to overcome the above disadvantages by combining them with biomass materials [21,22]. Chitosan (CS) is a biomass material with great potential for Cr(VI) wastewater treatment. On the one hand, CS is a naturally degradable polymer material widely obtained from animal carapaces [23]. In addition, there are abundant amino and hydroxyl groups on the CS molecular chain that coordinate and chelate with heavy metal ions independently and assemble and modify with GO as well [20,24,25].

At present, using GO or CS as precursors and combining them with some materials has become an important way to develop high-performance Cr(VI) removal composites [16]. This can not only enrich the functional groups but also improve the stability and adsorption performance of composite materials [25,26,27]. For example, the research of Yaşar et al. (2009) showed that the maximum adsorption capacity of Cr(VI) on flake CS was 7.94 mg/g [28]. Melvin et al. (2019) used a CS-grafted GO nanocomposite to remove Cr(VI) [29]. The adsorption capacity for Cr(VI) was as high as 104.16 mg/g. Singh et al. (2022) prepared a novel nanocomposite by immobilizing CaO nanoparticles on the surface of GO, and the adsorption capacity of Cr(VI) was 38.04 mg/g [30]. Sheth et al. (2022) prepared a novel composite (CS-[BMIM] [OAc]) by modifying the CS with 1-butyl-3-methylimidazolium acetate. The adsorption capacity of Cr(VI) was 125.63 mg/g [31]. In addition, iron-based materials have the advantages of high efficiency, environmental benignness, abundant resources, and easy magnetic separation, and they are potential candidate materials for adsorbents [32]. Recently, it has become common to add iron-based materials to CS or GO composites [25,33,34,35]. This type of composite can be rapidly separated by applying a magnetic field from the water after Cr(VI) adsorption, thus reducing solid–liquid separation processes (such as centrifugation or filtration) and improving the secondary recovery efficiency [36,37,38]. For example, by supporting Fe_2_O_3_ on GO to prepare a reductive nanocomposite (rGO), the maximum adsorption capacity of Cr(VI) was 41.67 mg/g [35]. Compared with GO, rGO was much more easily separated from the solution after Cr(VI) removal. Daimei et al. (2013) had prepared CS/montmorillonite–Fe_3_O_4_ microsphere to remove Cr(VI) [39]. This microsphere showed an adsorption capacity of 58.82 mg/g (higher than the mean value of CTS and MMT) and retained good removal efficiency after reusing over 3 cycles. Chaoke et al. (2020) prepared α–Fe_2_O_3_/GO nanocomposite [40], and its adsorption capacity and removal rate were 172.63 mg/g and 86.31%, respectively. The CS/magnetite–GO (MGO) composite was prepared by depositing Fe_3_O_4_ particles on the surface of the GO to fabricate the MGO hybrid first and then attaching CS on MGO sheets [16]. This composite showed an adsorption capacity of 120.97 mg/g for Cr(VI) (higher than that of CS, GO/CS and glutaraldehyde cross-linked CS). Shan et al. (2021) applied a GOCS composite (Fe_2_O_3_–GOCS) modified by Fe_2_O_3_ to remove Cr(VI) from wastewater [25]. It was found that the adsorption capacity of Fe_2_O_3_–GOCS was 72.53 mg/g, higher than 52.03 mg/g of GOCS. The zero-valent iron GO alginate beads were prepared by immobilization of pre-synthesized zero-valent iron nanoparticles into GO-modified alginate gel [41]. The introduction of zero-valent iron could raise the Cr(VI) removal efficiency of GO–alginate beads from 55.1% to 87.0%. The above studies indicate that iron-based materials, such as magnetite, ferric oxide, and zero-valent iron [25,39,41], can further improve the adsorption capacity of GO or CS composites for Cr(VI), and that different types of iron modify GO or CS composites have different effects on the adsorption capacity of Cr(VI). Additionally, goethite (α–FeO(OH)) has been proven to be another type of iron-based material with excellent adsorption performance due to its stability, abundance, and low cost [20,42,43]. However, to the best of our knowledge, modifying the GOCS composite with α–FeO(OH) to prepare α–FeO(OH)/GOCS microsphere for researching the performance and mechanism of Cr(VI) adsorption has not been reported. In addition, few studies have noticed the potential danger of secondary pollution caused by the Fe dissolution of iron-based adsorbents [20,25].

Therefore, this study was an attempt to investigate the performance and mechanism of α–FeO(OH)/GOCS microsphere as an adsorbent for Cr(VI) removal from aqueous solutions and evaluate its possibility of Fe secondary pollution. The characterization was conducted by X-ray powder diffraction (XRD), Fourier transform infrared spectroscopy (FT-IR), Scanning Electron Microscopy with Energy Dispersive Spectrometer (SEM-EDS), and X-ray photoelectron spectroscope (XPS) analysis. Batch experiments were carried out to determine the effects of pH, mass, and volume ratio (*m*/*v*), coexisting ions, time (*t*), temperature (*T*), and Cr(VI) initial concentration (*C*_0_) on Cr(VI) removal, and to evaluate the possibility of Fe secondary pollution during the microspheres adsorption for Cr(VI). Kinetic, isotherm, and thermodynamic characteristics were studied to further understand the adsorption mechanisms.

## 2. Materials and Methods

### 2.1. Materials

GO with a purity of 95 wt% was purchased from Tanfeng Graphene Technology Co., Ltd. (Suzhou, China). CS with deacetyiation of more than 90% and glutaraldehyde with a purity of 50% were purchased from Lanji Technology Development Co., Ltd. (Zhuhai, China), and Aladdin Reagent Co., Ltd. (Shanghai, China), respectively. Other AR-grade reagents and chemicals, including HCl, FeCl_3_·6H_2_O, K_2_Cr_2_O_7_, anhydrous ethanol, NaOH, acetic acid, and methanol, were purchased from Xilong Science Co., Ltd. (Shanghai, China). Ultrapure water was prepared using a Milli–Q water system (Millipore, Boston, MA, USA).

### 2.2. Synthesis of α–FeO(OH) Microspheres

Referring to the reported method used for preparing goethite [25], α–FeO(OH)/GOCS microspheres were prepared as follows: a total of 200 mL of 1.5% acetic acid solution was prepared, 0.8 g of GO powder was added, and the solution was stirred ultrasonically until the GO powder was uniformly dissolved. The GOCS solution was prepared after the CS powder was uniformly dissolved. Then, 20.7 g FeCl_3_·6H_2_O was added to the GOCS solution and stirred until it was uniformly dissolved to obtain the α–FeO(OH)/GOCS mixture. Using 10 mL syringes, the α–FeO(OH)/GOCS mixture was dropped into a 400 mL 20% NaOH solution at a constant rate to form microspheres evenly. The α–FeO(OH)/GOCS microspheres in the aqueous solution were left standing for 24 h and then repeatedly soaked in ultrapure water to wash the pH of the solution to neutral. Then, filtration, adding methanol-glutaraldehyde solution, standing curing crosslinking for 2.5 h. The solution was fully washed with anhydrous ethanol, filtered, and dried to obtain α–FeO(OH)/GOCS microspheres. The microsphere was named α–FeO(OH)/GOCS because the form of iron in this composite was α–FeO(OH) from the following XRD analysis.

### 2.3. Batch Adsorption Experiment

The batch adsorption experiments aimed to explore the effects of pH (3.0–11.0), mass, and volume ratio (*m*/*v*) (0.2–1.4 g/L), coexisting ions (0.1 mmol/L, 1 mmol/L, and 10 mmol/L Cl^−^, NO_3_^−^, SO_4_^2−^, PO_4_^3−^, Ca^2+^, Mg^2+^ and Mn^2+^), time (*t*) (5–3840 min), temperature (*T*) (298.15 K, 308.15 K, and 318.15 K) and Cr(VI) initial concentration (*C*_0_, 5–300 mg/L). Then, further sorption experiments were conducted to characterize adsorption kinetics, isotherms, and thermodynamics at the optimum values of pH and *m*/*v*. The α–FeO(OH)/GOCS microspheres were added to a series of 50 mL Cr(VI) solutions with 25 mg/L, and their pH was adjusted by 0.1 mol HCl and NaOH. Then, the mixtures were shaken until equilibrium was reached. Finally, the supernatant was collected for Cr(VI) concentration measurement.

All experiments were performed in 100 mL PE tubes at a shaking rate of 170 rpm, and three repetitions were performed to obtain repeatable results with an error of less than 5%. All aqueous samples were filtered with a 0.45 μm filter before the Cr(VI) concentration was determined.

The equilibrium adsorption capacity (*q_e_*) and removal rate (*r_e_*) of α–FeO(OH)/GOCS microspheres for Cr(VI) were calculated by the formulas below:

(1) $$q_{e}=\frac{\left(C_{0}-C_{e}\right)\cdot V}{m}$$

(2) $$r_{e}=\frac{\left(C_{0}-C_{e}\right)}{C_{0}}\times 100\%$$

where *q_e_* is the adsorption capacity of α–FeO(OH)/GOCS for Cr(VI) at adsorption equilibrium, mg/g; *C*_0_ is the Cr(VI) initial concentration, mg/L; *C_e_* is the concentration of Cr(VI) at adsorption equilibrium, mg/L; *V* is the volume of simulated liquid containing Cr(VI), L; *m* is the dosage of adsorbent, g; *r_e_* is the removal rate of Cr(VI) at adsorption equilibrium, %.

### 2.4. Kinetic, Isotherm, and Thermodynamic Models

The pseudo-first-order kinetic model, pseudo-second-order kinetic model, and Weber–Morris model were used to fit the adsorption of α–FeO(OH)/GOCS for Cr(VI). The equations associated with the above three models were listed as the following Equations (3)–(5):

(3) $$log\left(q_{e}-q_{t}\right)=log\;q_{e}-\frac{K_{1}}{2.303}t$$

(4) $$\frac{t}{q_{t}}=\frac{1}{K_{2}q_{e}^{2}}+\frac{t}{q_{e}}$$

(5) $$q_{t}=k_{di}t^{\frac{1}{2}}+c_{i}$$

where *q_e_* and *q_t_* are the removal capacity of Cr(VI) at adsorption equilibrium and time (*t*) respectively, mg/g; *K*_1_ and *K*_2_ are the adsorption rate constants of pseudo-first-order and pseudo-second-order kinetics, respectively; *K_di_* is the diffusion rate constant within the particle; *C_i_* is a constant related to boundary layer thickness.

The Langmuir, Freundlich, and Sips models were used to study the adsorption mechanism and compute the adsorption-related parameters. The three models were as follows:

(6) $$\mathrm{Langmuir}\;:q_{e}=\frac{q_{m}K_{L}C_{e}}{1+K_{L}C_{e}}$$

(7) $$\mathrm{Freundlich}:q_{e}=K_{F}C_{e}^{1/n}$$

(8) $$\mathrm{Sips}:\;q_{e}=\frac{q_{m}K_{s}C_{e}^{1/m}}{1+K_{s}C_{e}^{1/m}}$$

where *q_e_* is the adsorption capacity of α–FeO(OH)/GOCS for Cr(VI) at adsorption equilibrium, mg/g; *C_e_* is the mass concentration for Cr(VI) in the solution at adsorption equilibrium, mg/L; *q_m_* is the maximum adsorption capacity of α–FeO(OH)/GOCS for Cr(VI), mg/g; *K_L_* is the Langmuir adsorption equilibrium constant and is related to the strength of the adsorption interaction; *K_F_* and 1/*n* are the adsorption equilibrium constant and adsorption strength constant of the Freundlich equation, respectively; *K_s_* is the adsorption equilibrium constant of the Sips equation; 1/*m* is used to measure the heterogeneity of the adsorption sites on the surface of the material. When the 1/*m* value is closer to 1, the adsorption sites on the adsorbent surface are more uniform [44].

Thermodynamic parameters were calculated using the following equations:

(9) $$\Delta G^{\theta }=\Delta H^{\theta }-T\Delta S^{\theta }$$

(10) $$K_{C}=\frac{(C_{0}-C_{e})\cdot V}{c_{e}\cdot m}$$

(11) $$lnK_{c}=-\left(\frac{\Delta H^{\theta }}{RT}\right)+\frac{\Delta S^{\theta }}{R}$$

where Δ*G^θ^* is the change in Gibbs free energy, kJ/mol; *T* is the temperature measured in Kelvin, K; *R* is the universal gas constant, 8314 J/(mol·K); *K_c_* is the thermodynamic equilibrium constant; *C*_0_ and *C_e_* are the initial and adsorption equilibrium mass concentrations of Cr(VI), respectively, mg/L; *V* is the volume of simulated liquid containing Cr(VI), L; *m* is the dosage of adsorbent, g; Δ*H^θ^* is the standard enthalpy change, kJ/mol; Δ*S^θ^* is the standard entropy change, J/(mol·K).

### 2.5. Analytical Techniques

The concentration of Cr(VI) in aqueous solutions was determined by an inductively coupled plasma optical emission spectrometer (Optima 7000DV, Platinum Elmer Instruments, Inc. Waltham, MA, USA), and the relative standard deviation was less than 5%. Microspheres before and after adsorption were used for characterization, including XRD, FT-IR, SEM-EDS, and XPS. The crystal structure was determined by X’Pert3 Powder-type multifunctional XRD (Panaco, Cu target, λ = 1.54056 Å, Almelo, The Netherlands). The scanning step, speed, and range were 0.02626°, 0.6565°/s, and 5–90° (2*θ*), respectively. The functional groups were measured using IS10 FT-IR (Thermo Fisher Scientific, Waltham, MA, USA). Surface morphology and elemental analysis were determined using SEM-EDS (JSM–7900F, JEOL, Tokyo, Japan). The chemical and electronic states of the elements were analyzed using XPS (WISDOM–9600, Shanghai, China). The Zeta potential of α–FeO(OH)/GOCS microspheres in an aqueous dispersion was measured by the Zetasizer Nano ZS analyzer (MALVERN, Nano zs90, Melvin, UK).

## 3. Results and Discussion

### 3.1. Material Characterization

#### 3.1.1. XRD

**Figure 1:**
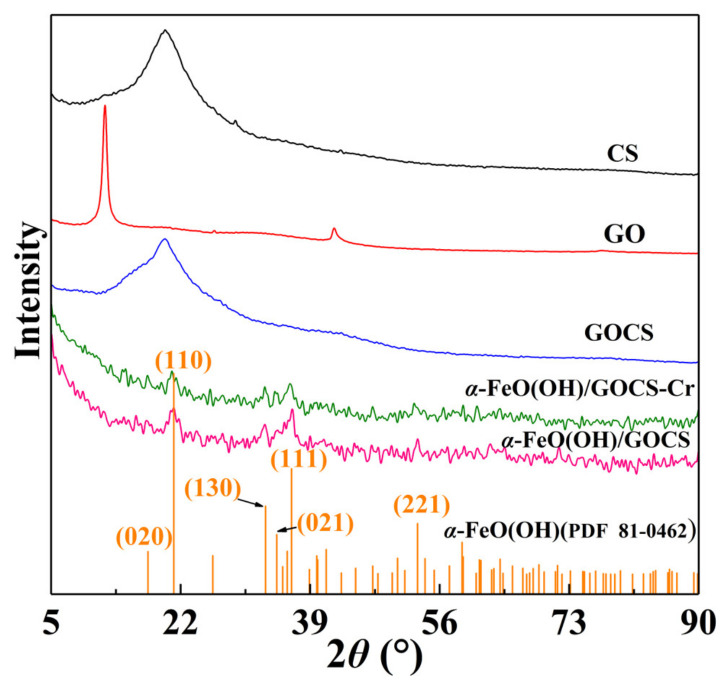
XRD patterns of GO, CS, GOCS, α–FeO(OH)/GOCS before Cr(VI) adsorption, and that of α–FeO(OH)/GOCS after adsorption for Cr(VI).

Figure 1 shows the XRD patterns of GO, CS, GOCS, and α–FeO(OH)/GOCS before and after Cr(VI) adsorption. GO had two diffraction peaks at 2*θ* = 12.19° and 2*θ* = 42.26°, and CS showed a diffraction peak at 2*θ* = 20.06°. The diffraction peaks of GOCS were at 2*θ* = 20.20°, and the peaks became wider and weaker. This showed that GOCS was successfully synthesized at room temperature [21]. After the addition of FeCl_3_·6H_2_O, new diffraction peaks of α–FeO(OH)/GOCS were at 2*θ* = 21.13° (110), 32.50° (130), 34.58° (021), 36.47° (111), and 53.06° (221). This indicated that the iron-bearing mineral in the adsorbent was goethite, and the form of iron was α–FeO(OH) by comparing it with the standard ICDD PDF card (No. 81–0462). Compared with the XRD patterns of α–FeO(OH)/GOCS before the adsorption of Cr(VI), the diffraction peaks of α–FeO(OH) became weaker after adsorption, indicating that α–FeO(OH) was involved in Cr(VI) adsorption.

#### 3.1.2. FT-IR

**Figure 2:**
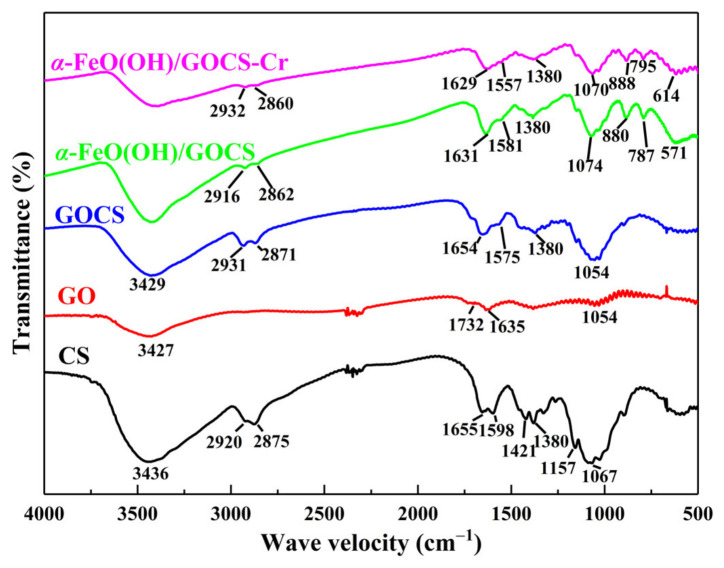
FT-IR spectra of GO, CS, GOCS, α–FeO(OH)/GOCS before Cr(VI) adsorption, and that of α–FeO(OH)/GOCS after adsorption for Cr(VI).

The spectra of GO, CS, GOCS, and α–FeO(OH)/GOCS before and after Cr(VI) adsorption are shown in Figure 2. The characteristic bands at 1067 cm^−1^ and 1157 cm^−1^ were related to the C–O bending vibration in CS [45] and at 1380 cm^−1^ and 1421 cm^−1^ were the C–H bending vibration bands in CH_2_, except for the water molecule vibration band at 3436 cm^−1^. The bands of 1655 cm^−1^ and 1598 cm^−1^ were the C = O stretching vibration bands of the amide I band, and at 2875 cm^−1^ and 2920 cm^−1^ were the C–H stretching vibration bands in CH_3_ and CH_2_ [46]. The characteristic bands at 1054 cm^−1^, 1635 cm^−1,^ and 1732 cm^−1^ were the bending vibration band of C–O–C, the bending vibration band of –OH, and the stretching vibration band of C = O in –COOH, respectively [47]. The spectra of GOCS retained some characteristic bands of GO and CS and had new characteristic bands. The new characteristic bands at 1654 cm^−1^ were the C = O stretching vibration band of amide –CONH–. The bands at 1575 cm^−1^ were deformation characteristic bands in the –NH_2_ plane. The intensity of the deformation characteristic bands was weakened, which may be related to the interference of –NH_2_ by the extremely strong characteristic band value of the amide bond [21]. The above results showed that GO and CS were successfully assembled to form GOCS and had abundant functional groups.

Compared with the spectra of GOCS, the spectra of α–FeO(OH)/GOCS showed that the bending vibration band of –OH moved to 1631 cm^−1^, and the band intensity decreased. In addition, the in-plane deformation characteristic band of –NH_2_ moved to 1581 cm^−1,^ and the band intensity of the bending vibration band of C–O–C decreased. These results suggest that –OH, –NH_2,_ and C–O–C were involved in the modification reactions of GOCS using α–FeO(OH). Meanwhile, the weak band at 571 cm^−1^ was the Fe–O vibration band, and the bands at 880 cm^−1^ and 787 cm^−1^ were the in-plane and out-of-plane bending vibration bands of –OH [21]. After adsorption for Cr (VI), the band of the –OH bending vibration at 1631 cm^−1^, the C–OH bending vibration at 1074 cm^−1^_,_ and the bending vibration bands of C–H at 1380 cm^−1^ became weakened, and the location of the Fe–O band shifted to 614 cm^−1^. These results indicated that –OH, C–OH, C–H, and Fe–O participated in Cr(VI) removal.

#### 3.1.3. SEM-EDS

**Figure 3:**
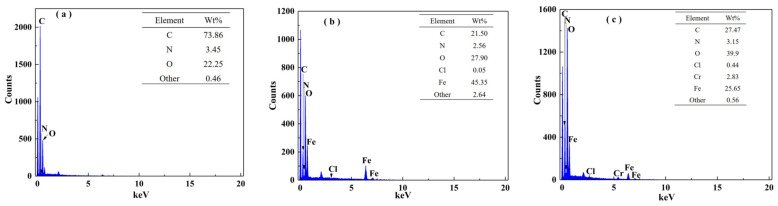
EDS spectra of GOCS (**a**), α–FeO(OH)/GOCS before (**b**) and after (**c**) Cr(VI) adsorption.

The SEM images of GOCS and α–FeO(OH)/GOCS are shown in Figure S1 in the Supplementary Materials. The surface of the GOCS was randomly distributed, with various fragments and abundant pores. After α–FeO(OH) modification, some needle-like substances appeared on the surface of the GOCS. These needle-like substances are intertwined to make many orderly folds. After Cr(VI) adsorption, the needle-like substances were replaced with some dispersedly scaly or flaky substances, which may have been chromium adsorbed on the α–FeO(OH)/GOCS surface. Figure 3 shows the EDS results of GOCS, α–FeO(OH)/GOCS before and after Cr(VI) adsorption. The major elements of GOCS were C, O, and N (Figure 3a). After the addition of FeCl_3_·6H_2_O, Fe and Cl appeared, and the counts of O also increased, indicating that GOCS was well modified by α–FeO(OH) (Figure 3b). The result was consistent with the XRD analysis of α–FeO(OH)/GOCS characteristic peaks. The existence of chromium confirmed that Cr(VI) was adsorbed to the surface of the α–FeO(OH)/GOCS (Figure 3c). In addition, the decrease in Fe content from 45.35% to 25.65% after Cr(VI) adsorption was mainly related to the dissolution of α–FeO(OH) in an acid environment (see Figure S2, Supplementary Materials). The increase in O content from 27.90% to 39.9% was related to the fact that Cr(VI) was reduced to Cr(III) by a reductive hydroxyl group (C–OH), and the produced Cr(III) was precipitated as Cr(III) oxides (Cr_2_O_3_ or Cr(OH)_3_) on the surface of the α–FeO(OH)/GOCS.

#### 3.1.4. XPS

**Figure 4:**
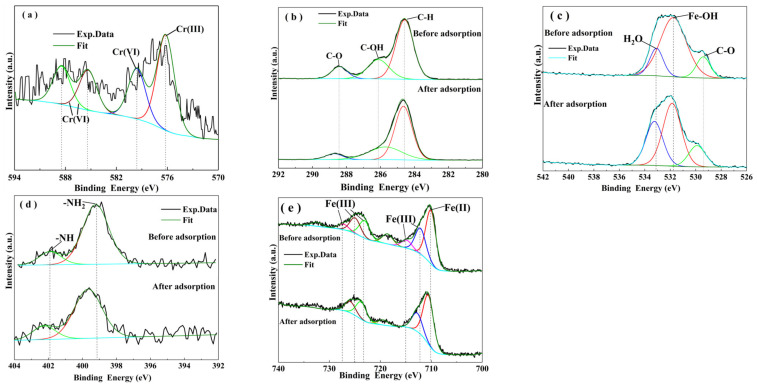
The XPS response spectra of Cr2p (**a**) after Cr(VI) adsorption, and C1s (**b**), O1s (**c**), N1s (**d**), and Fe2p (**e**) before and after Cr(VI) adsorption.

Figure 4 shows the XPS scanning results of α–FeO(OH)/GOCS before and after Cr(VI) adsorption. After Cr(VI) adsorption, four new peaks appeared at binding energies of 576.62 eV and 579.58–588.45 eV, respectively, as shown in Figure 4a. The former belonged to Cr(III) in the form of Cr_2_O_3_ or Cr(OH)_3_ [48,49], and the latter belonged to Cr(VI) in the form of HCrO_4_^−^ and Cr_2_O_7_^2−^ [35]. These results showed that the adsorbed Cr(VI) was partly reduced to Cr(III) during α–FeO(OH)/GOCS adsorption for Cr(VI). Similar results have been reported for Cr(VI) removal using an iron-based modified GOCS composite [25,35]. Cr(III) was not found in the aqueous solution after Cr(VI) adsorption. This indicates that Cr(VI) was first adsorbed to α–FeO(OH)/GOCS. Then, the adsorbed Cr(VI) was partly reduced to Cr(III), which precipitated in the form of Cr_2_O_3_ or Cr(OH)_3_ on the surface of α–FeO(OH)/GOCS [25]. Another adsorption, Cr(VI), mainly existed in the form of an ionic complex on the adsorbent by electrostatic attraction. The C1s spectra of α–FeO(OH)/GOCS before and after Cr(VI) adsorption are shown in Figure 4b. The peaks at the binding energies of 284.65 eV, 285.70 eV, and 288.72 eV were C–H, C–OH, and C–O, respectively [50]. After Cr(VI) adsorption, the peaks of C–H, C–OH, and C–O were weakened and shifted to a higher binding energy. Similar results were also found in the change in the Fe–OH peak from the O1s spectra in Figure 4c. These results indicated that C–H, C–OH, C–O, and Fe–OH had been involved in Cr(VI) adsorption and were consistent with the results of the FT-IR analysis. However, almost nothing changed in the N1s spectra after adsorption (Figure 4d), indicating that the functional groups of –NH_2_ did not participate in Cr(VI) adsorption. Figure 4e shows that Fe(III) and Fe(II) coexisted in the microspheres. Before Cr(VI) adsorption, the peaks at binding energies of 710.10 eV and 712.16–727.26 eV were Fe(II) and Fe(III), respectively [50,51]. After adsorption for Cr(VI), there was no change in the peak of Fe(II), indicating that Fe(II) was not involved in Cr(VI) adsorption. The peaks of Fe(III) at 714.69 eV and 727.26 eV disappeared. These were attributed to the dissolution of α–FeO(OH) in an acid environment and corresponded to the decreasing Fe content in the EDS analysis. In addition, another intensity of Fe(III) peaks became weaker, indicating that some Fe(III) was involved in Cr(VI) adsorption.

### 3.2. Influencing Factors

#### 3.2.1. Effect of pH

**Figure 5:**
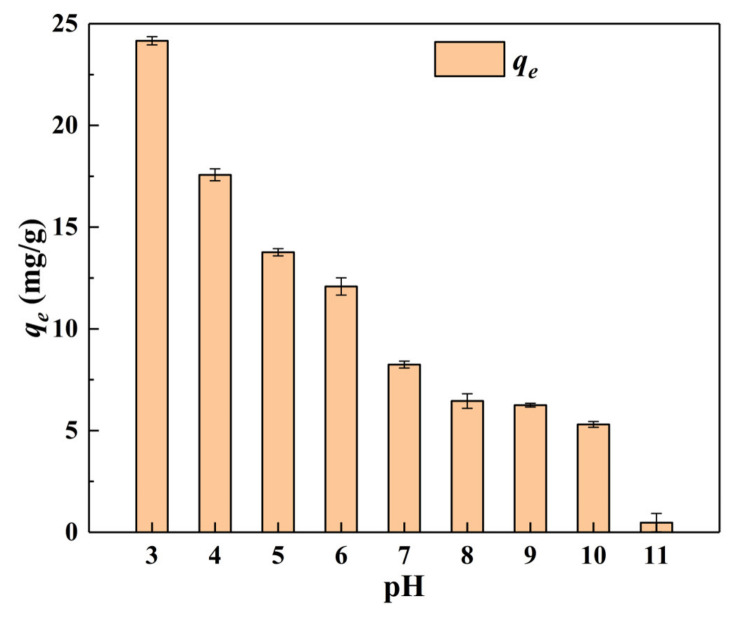
Changes in equilibrium adsorption capacity of Cr(VI) with pH value.

Figure 5 shows the equilibrium adsorption capacity (*q_e_*) of α–FeO(OH)/GOCS adsorption for Cr(VI), varying with pH values at *C*_0_ = 25 mg/L, *m*/*v* = 1.0 g/L, and *T* = 298.15 K. The *q_e_* values decreased with increasing pH values from 3.0 to 11.0. The *q_e_* value was the highest (24.16 mg/g) and the lowest (0.47 mg/g) when pH = 3 and 11, respectively, indicating that the pH values had a great influence on the *q_e_* of Cr(VI) adsorption. The experimental results were consistent with many studies on GOCS-based materials for Cr(VI) removal [25,52].

This could be explained by the distribution of chromium ions in the solution and the types of charged charges on the adsorbent surface [53]. When the solution was under acidic conditions, Cr(VI) mainly occurred in the ion forms of HCrO_4_^−^ and Cr_2_O_7_^2−^. Under alkaline conditions, it primarily exists in the form of CrO_4_^2−^ [5]. In addition, the pH at the potent of zero charges (*pH_pzc_*) for α–FeO(OH)/GOCS was 8.2 [20]. This indicated that the surface of α–FeO(OH)/GOCS had positive charges when the pH value was below 8.2 and negative charges at a pH value above 8.2. Therefore, Fe–OH on the surface of α–FeO(OH)/GOCS was protonated to Fe–OH_2_^+^ when the pH value was 3. Then, the electrostatic attraction was improved between Fe–OH_2_^+^ and HCrO_4_^−^ (or Cr_2_O_7_^2−^), making Cr(VI) adsorbed to a large number of surface sites. Meanwhile, the partly adsorbed Cr(VI) could be reduced to Cr(III) by the reductive hydroxyl groups (C–OH) of the microspheres, and then the reduced Cr(III) in the form of Cr_2_O_3_ or Cr(OH)_3_ precipitated on the surface of the microspheres [25]. The OH^−^ concentration and –OH_2_^+^ deprotonation increased with increasing pH, and then the electrostatic repulsion was improved between them and CrO_4_^2−^, causing the *q_e_* to decrease significantly.

#### 3.2.2. Effect of *m*/*v*

**Figure 6:**
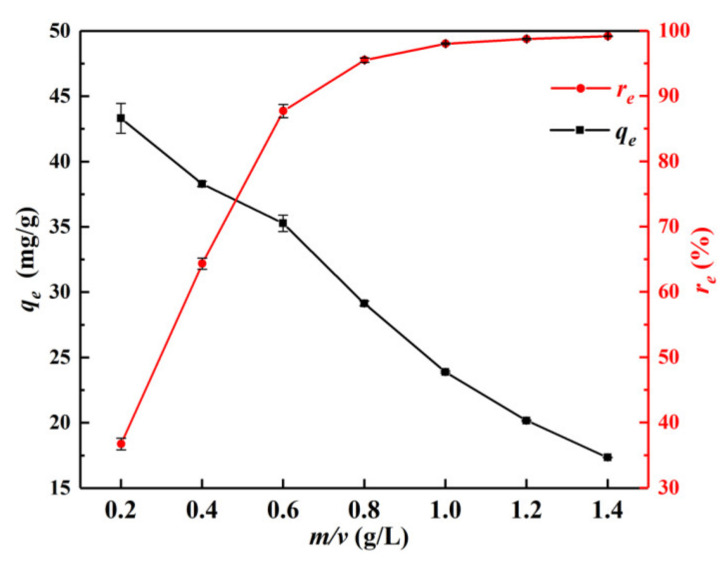
Changes in equilibrium adsorption capacity (*q_e_*) and removal rate (*r_e_*) of Cr(VI) with *m*/*v* values.

Figure 6 shows the changes in the equilibrium adsorption capacity (*q_e_*) and the removal rate (*r_e_*) of Cr(VI) on α–FeO(OH)/GOCS with the *m*/*v* values under the conditions of *C*_0_ = 25 mg/L, pH = 3.0, and *T* = 298.15 K. It can be seen that the *r_e_* value of Cr(VI) increased from 36.71% to 99.15%, and the *q_e_* value decreased from 43.31 mg/g to 17.35 mg/g with increasing *m*/*v*. The *r_e_* value rose rapidly when the *m*/*v* values increased from 0.2 g/L to 0.6 g/L. This may be related to the fact that the effective adsorption sites on the surface of the microsphere were limited. Therefore, the *r_e_* value can rapidly increase with the increasing *m*/*v* value while the *q_e_* value decreases. After that, the *r_e_* value increased slowly with the *m*/*v* value increasing from 0.6 g/L to 1.4 g/L, indicating that the adsorption of Cr(VI) by α–FeO(OH)/GOCS gradually reached saturation. To achieve a better effect of Cr(VI) removal, *m*/*v* around 1.0 g/L should be selected.

#### 3.2.3. Effect of Coexisting Ions

**Figure 7:**
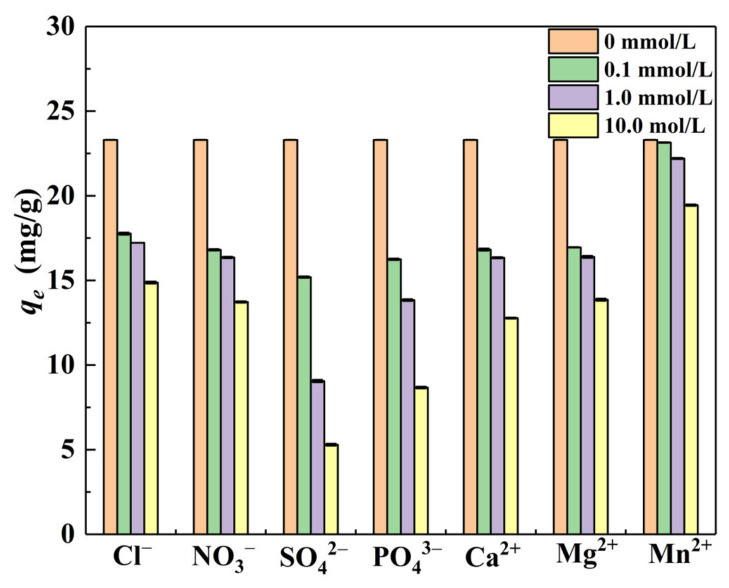
Effects of coexisting ions on Cr(VI) equilibrium adsorption capacity (*q_e_*).

Cl^−^, NO_3_^−^, SO_4_^2−^, PO_4_^3−^, Ca^2+^, Mg^2+^_,_ and Mn^2+^ are selected to study the influence on the adsorption of Cr(VI) in an aqueous solution. The experimental results are shown in Figure 7. The initial concentrations of all ions were 0.1 mmol/L, 1.0 mmol/L, and 10.0 mmol/L, respectively. Other reaction conditions included *C*_0_ = 25 mg/L, pH = 3.0, *m*/*v* = 1.0 g/L, and *T* = 298.15K. It can be seen that the higher the concentration of cations and anions, the stronger the inhibition ability of Cr(VI) removal. The degree of influence of the anions on the *q_e_* value of Cr(VI) in descending order was SO_4_^2−^ > PO_4_^2−^ > NO_3_^−^ > Cl^−^, while the influence degree of cations on the *q_e_* value of Cr(VI) in descending order was Ca^2+^ > Mg^2+^ > Mn^2+^. The *q_e_* value was 23.30 mg/g under no ions were added. When SO_4_^2−^ was added at 0.1 mmol/L, 1.0 mmol/L and 10.0 mmol/L, the *q_e_* value decreased by 8.10 mg/g, 14.24 mg/g and 18.01 mg/g, respectively. Then the *q_e_* value decreased by 6.48 mg/g, 6.96 mg/g and 10.52 mg/g, respectively under the influence of Ca^2+^. This was because anions could compete with Cr(VI) anions in the main forms of HCrO_4_^−^ and Cr_2_O_7_^2−^ for the active sites on the adsorbent, and α–FeO(OH) likewise displayed a strong adsorption capacity for SO_4_^2−^ and PO_4_^2−^ [54]. In addition, the positive charges were distributed on the surface of the material, and there was a strong repulsive force with these cations (Ca^2+^, Mg^2+^, and Mn^2+^) under the acidic conditions. Other researchers have reported similar results [25,52].

#### 3.2.4. Effect of Contact Time

**Figure 8:**
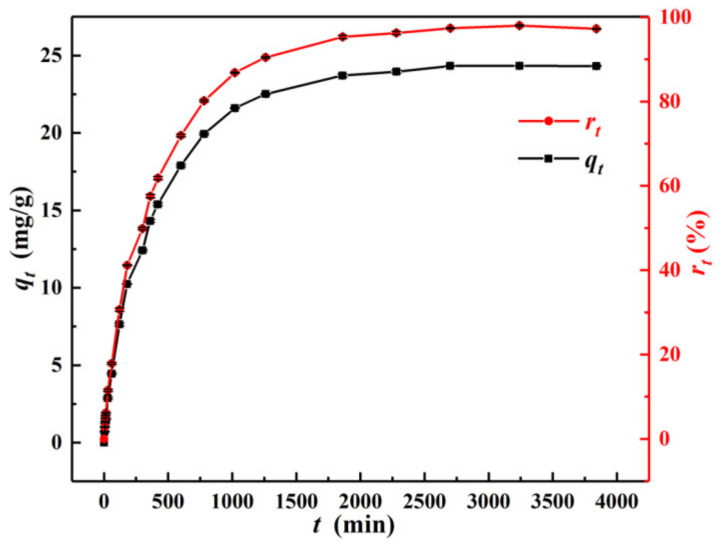
Cr(VI) adsorption amount (*q_t_*) and removal rate (*r_t_*) changes with the increasing contact time at 298.15 K, respectively.

Figure 8 shows the changes in the adsorption amount (*q_t_*) and removal rate (*r_t_*) of Cr(VI) with contact time (*t*) at *C*_0_ = 25 mg/L, pH = 3.0, *m*/*v* = 1.0 g/L, and *T* = 298.15 K. It showed that the *q_t_* and *r_t_* values both rapidly increased before 180 min. This was because α–FeO(OH)/GOCS had abundant adsorption sites and pores at the beginning, and it swiftly adsorbed Cr(VI) under strong electrostatic attraction [52]. Then *q_t_* and *r_t_* values increased slowly between 300 min and 1260 min due to the saturation of adsorption sites and pores on the surface. From 1860 min to 3840 min, the *q_t_* and *r_t_* values reached 24.32 mg/g and 97.78%, respectively. In this stage, the adsorption sites on the surface of the material reached saturation, suggesting that the reaction reached equilibrium. Therefore, more than 1860 min was determined as the time of the equilibrium stage.

#### 3.2.5. Effect of Initial Concentration

**Figure 9:**
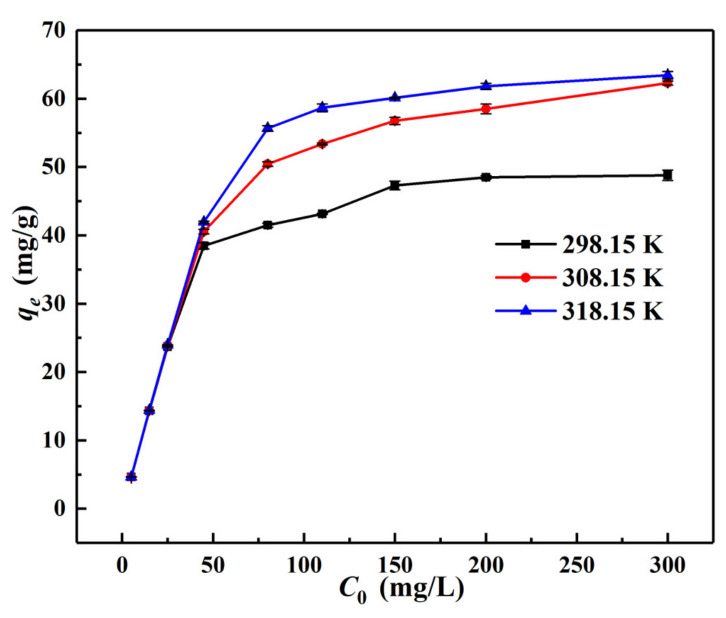
Influence of initial concentration on Cr(VI) adsorption by α–FeO(OH)/GOCS.

Figure 9 shows the changes of equilibrium adsorption capacity (*q_e_*) with initial concentration (*C*_0_, 5–300 mg/L) under the conditions of pH = 3.0, *m*/*v* = 1.0 g/L and *T* = 298.15 K, 308.15 K, and 318.15 K, respectively. This showed that the *q_e_* value increased promptly first and then gently later until it became balanced with the increase of *C*_0_. This may indicate that Cr(VI) adsorption on α–FeO(OH)/GOCS was a diffusion-driven process [25]. At 298.15 K, 308.15 K, and 318.15 K, the *q_e_* value reached 48.80 mg/g, 62.30 mg/g, and 63.43 mg/g respectively, indicating that elevating the temperature could increase the *q_e_* value of Cr(VI). This was probably because increasing temperatures can strengthen the irregular movement of Cr(VI) ions and enhance the pore size expansion and surface activation of the adsorbent to adsorb more Cr(VI) [55].

### 3.3. Adsorption Characteristics

#### 3.3.1. Adsorption Kinetics

The pseudo-first-order kinetic model assumed that adsorption was predominantly physical [56], while the pseudo-second-order kinetic model assumed that adsorption was mainly chemical [57]. The Weber-Morris model facilitates the understanding of the mechanism described by external mass transfer (boundary layer diffusion) and intraparticle diffusion [58].

**Figure 10:**
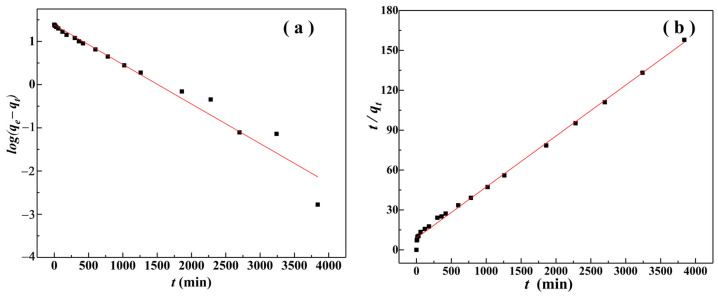
Pseudo-first-order kinetic plot (**a**) and pseudo-second-order kinetic (**b**) plot for Cr (VI) adsorption on α–FeO(OH)/GOCS at 298.15 K.

Figure 10 and Table 1 show the fitting analysis results of the pseudo-first-order kinetic model and pseudo-second-order kinetic model to the experimental data. It can be seen that under the conditions of *C*_0_ = 25 mg/L, pH = 3, *m*/*v* = 1.0 g/L, and *T* = 298.15 K, the determination coefficient (*R*^2^) of the pseudo-second-order kinetics was greater than 0.99, indicating that the Cr(VI) adsorption was more consistent with the pseudo-second-order kinetic model and was mainly a chemical mode of adsorption [30].

**Figure 11:**
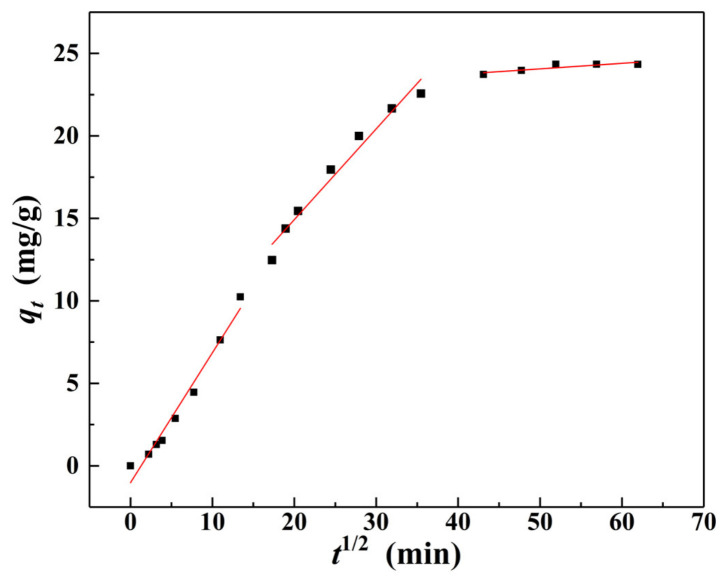
Fitting curve of the Weber-Morris model for Cr (VI) adsorption by α–FeO(OH)/GOCS at 298.15 K.

Figure 11 and Table 2 show the adsorption results of the Weber–Morris model. The fitting results were consistent with the changes in adsorption amount (*q_t_*) and removal rate (*r_e_*) of Cr(VI) with contact time (*t*) (Figure 8), and the fitting of adsorption of Cr(VI) could be divided into three stages. In the first stage, *q_t_* value rapidly increased with the increase of *t^1^*^/*2*^. This was because the surface of α–FeO(OH)/GOCS had abundant adsorption sites at the initial stage of adsorption that could quickly combine with Cr(VI) in the solution. In this stage, α–FeO(OH)/GOCS mainly adsorbed Cr(VI) on the surface. The second stage was slow adsorption; the adsorption sites on the surface progressively attained saturation, and the adsorption mode switched from surface adsorption to pore diffusion. In the third stage, adsorption reached equilibrium because of the saturation of the adsorption sites. In addition, *q_t_* and *t^1^*^/*2*^ in each of the above three stages follow a linear function. However, such linear functions did not pass through the origin (meaning that *q_t_* did not drop to zero at *t* = 0), and *K_id_* kept decreasing and *C_i_* kept increasing. This indicated that Cr(VI) adsorption was not only affected by internal diffusion but may also be affected by the molecular size, concentration, affinity, and diffusion coefficient of adsorbate in the aqueous solution [59].

#### 3.3.2. Adsorption Isotherms

The Langmuir model assumed that the adsorbent had a uniform surface structure, dominated by monolayer adsorption, and that its binding sites had the same adsorption tendency and did not interact with each other [60]. The Freundlich model assumed that the adsorbent surface was inhomogeneous and considered multilayer adsorption [61]. The Sips adsorption isotherm model is a comprehensive model with asymptotic properties [62], and the suitable adsorption amount eventually reaches its maximum value. When the heterogeneity factor 1/*m* was equal to 1, indicating that the distribution of adsorption sites on the surface was uniform [44], then the Sips model was equivalent to the Langmuir isotherm [62].

**Figure 12:**
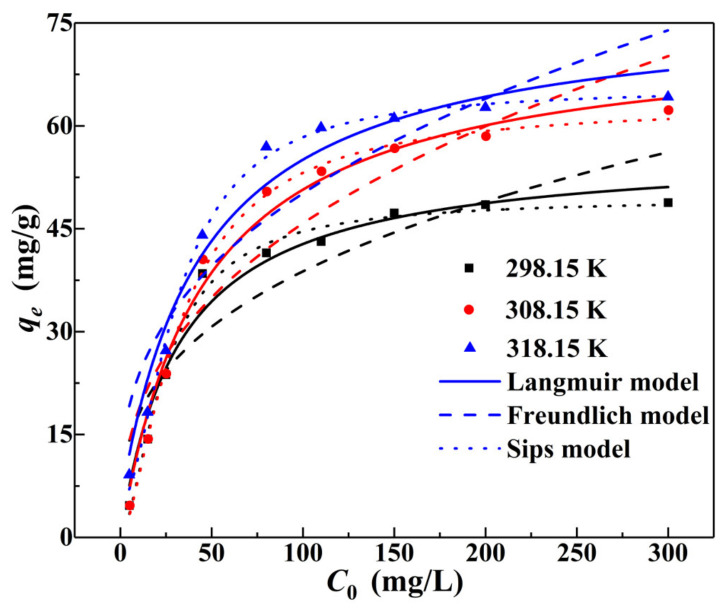
Adsorption isotherm fitting curves of Cr(VI) on the α–FeO(OH)/GOCS.

Figure 12 and Table 3 show the results of using the Langmuir, Freundlich, and Sips models to fit the experimental data on the adsorption of Cr(VI). The Langmuir model and Sips model fit the adsorption data best, and the determination coefficients (*R*^2^) were in the range of 0.9650–0.9804 and 0.9885–0.9964, respectively. When the heterogeneity factor (1/*m*) was equal to 1, indicating that the distribution of adsorption sites on the surface was uniform [44] and the Sips model was equivalent to the Langmuir isotherm [62]. Therefore, the best-fitted 1/*m* values of 1.51–1.76 in the Sips model indicated that the adsorption of Cr(VI) was mainly monolayer adsorption, with a certain heterogeneity. In addition, at 298.15 K, 308.15 K, and 318.15 K, the maximum adsorption capacities of Cr(VI) were 49.37 mg/g, 63.19 mg/g, and 64.77 mg/g, respectively, which were closer to the actual situation (Figure 9).

The basic features of the Langmuir model can be analyzed in terms of *R_L_*. *R_L_* was a dimensionless constant used to forecast whether an adsorption system was appropriate for adsorption [63]. Defined as follows:

(12) $$R_{L}=\frac{1}{1+K_{L}C_{0}}$$

where *C*_0_ is the initial concentration of Cr(VI), mg/L; *K_L_* is the Langmuir constant.

From the relationship between *C*_0_ and *R_L_* at different concentrations (5–300 mg/L) and temperatures (298.15 K, 308.15 K, and 318.15 K) (see Figure S3, Supplementary Materials), the *R_L_* value was lower if the initial concentration was higher, indicating that it was supportive of adsorption by increasing the initial concentration of Cr(VI) [63]. The value of *R_L_* was in the range of 0–1, suggesting that Cr(VI) adsorption was spontaneous [63].

#### 3.3.3. Thermodynamic Characteristics

**Figure 13:**
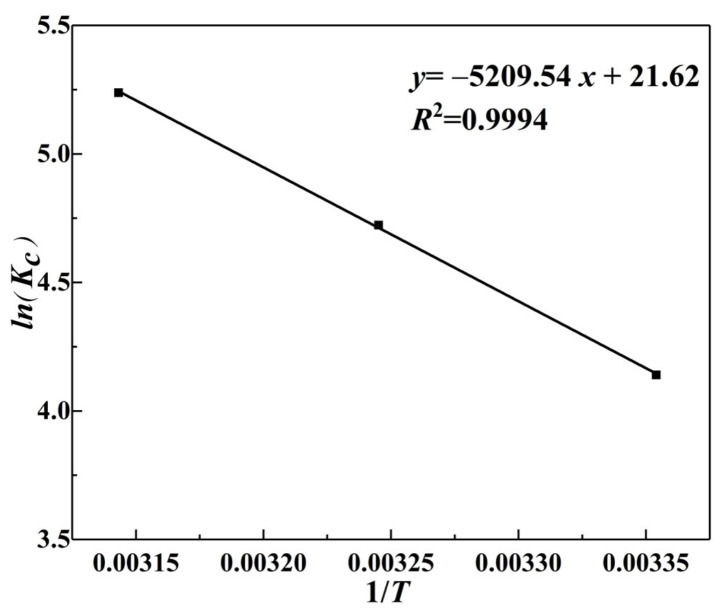
Van’t Hoff curve of α–FeO(OH)/GOCS adsorption of Cr(VI).

To analyze the effect of ambient temperature on the adsorption of Cr(VI) and the reaction process using thermodynamic parameters [64]. Figure 13 shows the linear equations of *lnK_c_* and 1/*T* calculated according to the experimental data, and Table 4 shows the thermodynamic parameters. This showed that Δ*H^θ^* was 43.31 kJ/mol, indicating that adsorption was an endothermic reaction. Then Δ*S^θ^* = 179.75 J/(mol·K) indicated that the adsorption of Cr(VI) was a large degree of disorder or randomness in the system. The Δ*G^θ^* values were negative at 298.15 K, 308.15 K, and 318.15 K, and the absolute value of Δ*G^θ^* increased with increasing temperature, indicating that the adsorption process of Cr(VI) was spontaneous. This result confirmed the previous results in Section 3.3.2 regarding the analysis of *R_L_*.

### 3.4. Performance Evaluation

Table 5 shows the comparison of Cr(VI) adsorption capacity between α–FeO(OH)/GOCS microsphere and other similar composites. The maximum adsorption capacities (*q_m_*) of α–FeO(OH)/GOCS for Cr(VI) were 63.43 mg/g (experimental data) and 63.19 mg/g (Sips model fitting result) under the conditions of *C*_0_ = 25 mg/L, pH = 3, *m*/*v* = 1.0 g/L. It could be seen that the *q_m_* value of Cr(VI) was higher than those for the most similar composite materials reported before, such as CS/magnetic bagasse biochar (8.78 mg/g) [65], magnetic glutaraldehyde cross-linked CS on nitrogen-doped GO (30.2 mg/g) [47], magnetite nanomaterials (34.87 mg/g) [66], fibrous mat of CS/polyvinyl alcohol/containing cerium(III) (52.88 mg/g) [67], magnetic CS (55.80 mg/g) [68] and Fe_3_O_4_ NPs/CS/glyoxal hydrogel (27.25 mg/g) [69], etc. These results indicate that α–FeO(OH)/GOCS microsphere had a good effect on Cr(VI) adsorption. Other similar chitosan-based composites, such as 1-butyl-3-methylimidazolium acetate modified CS (CS- [BMIM] [OAc], 125.63 mg/g) [31] and CS/cellulose nanocrystals/carbon dots composite (CNCD, 217.80 mg/g) [70], show a higher *q_m_* value than α–FeO(OH)/GOCS prepared in this study; these provided references for further optimization of the adsorption properties.

**Table 5:** Adsorption capacity of other adsorbents for Cr(VI).

Adsorbent	pH	Temperature (K)	*C*_0_ (mg/L)	*q_m_* (mg/g)	References
α–FeO(OH)/GOCS	3.0	308.15	5–300	63.19	This study
CS	4.0	308.15	5–200	7.94	[28]
Sea shell (CaO NPs) GO	3.3	303.00	10–50	38.04	[30]
Chitosan-[BMIM][OAc]	3.0	298.15	50–300	125.63	[31]
Reductive Fe_2_O_3_/GO (rGO) nanocomposite	2.0	288.00	12.66–37.65	41.67	[35]
Magnetic glutaraldehyde cross-linked CS on nitrogen-doped GO(MCC@NGO)	3.0	297.00	4	30.20	[47]
	2.0	297.00	2.7	15.90	
CMB(γ–Fe_2_O_3_)	2.0	298.15	10–100	8.78	[65]
Fe_3_O_4_ nanoparticle	–	328.15	2–100	34.87	[66]
Fibrous mat of CS/polyvinyl alcohol/containing cerium(III)	4.0	208.15	10–50	52.88	[67]
Magnetic CS	3.0	298.00	60–180	55.80	[68]
Fe_3_O_4_ NPs/CS/glyoxal hydrogel	4.0	298.00	5–30	27.25	[69]
CS/cellulose nanocrystals/carbon dots (CNCD) composite	2.0	298.15	20–120	217.80	[70]

It was worth noting that the α–FeO(OH)/GOCS microsphere also had abundant functional groups, such as C–O, C = C, –CH, –OH, –NH_2_, and Fe–O, so it could be expected to remove other metal ions, organic pollutants, dyes, etc. This will be verified in future studies. In addition, the prepared α–FeO(OH)/GOCS in this study exhibited a high Cr(VI) removal ability in acidic aqueous solution, but a lower Cr(VI) removal ability in alkaline aqueous solution. This may limit the application of α–FeO(OH)/GOCS microsphere.

The advantages of the adsorbent were not only reflected in its high adsorption capacity but also in its stability and environmental benignness. However, few researchers have noted this problem before [21,35,39,40,71]. The removal rate (*r_e_*) of Cr(VI) and Fe concentration of α–FeO(OH)/GOCS varied with pH, as shown in Figure S2, Supplementary Materials. It can be seen that the maximum amount of Fe concentration in α–FeO(OH)/GOCS was only 0.17 mg/L in the measured pH range, far lower than the World Health Organization (WHO) drinking water limit of 0.3 mg/L for Fe. In addition, the Fe concentration was only 0.14 mg/L at pH = 3, which was lower than that of Fe_2_O_3_/GOCS (0.179 mg/L) studied by Shan et al. (2021) [25]. When pH = 3, the *r_e_* value of Cr(VI) reached 97.69%. It is higher than those of α–Fe_2_O_3_/GO nanocomposite (86.31%) [40], magnetic greigite/biochar (MGBs) composites (93.00%) [72], MnO_2_/CS nanocomposite (94.21%) [73] and polyamine modified carbon nanotube (PA–CNT) adsorbents (95.00%) [74]. These results indicated that the adsorbent did not cause secondary pollution to the environment, and at the same time retained good stability and a high removal rate in a strongly acidic solution. In addition, GOCS (a) and α–FeO(OH)/GOCS (b) appeared uniformly spherical at room temperature, as shown in the real diagrams of GOCS, and α–FeO(OH)/GOCS (Figure S4, Supplementary Materials) provided favorable conditions for recycling.

### 3.5. Adsorption Mechanism

**Figure 14:**
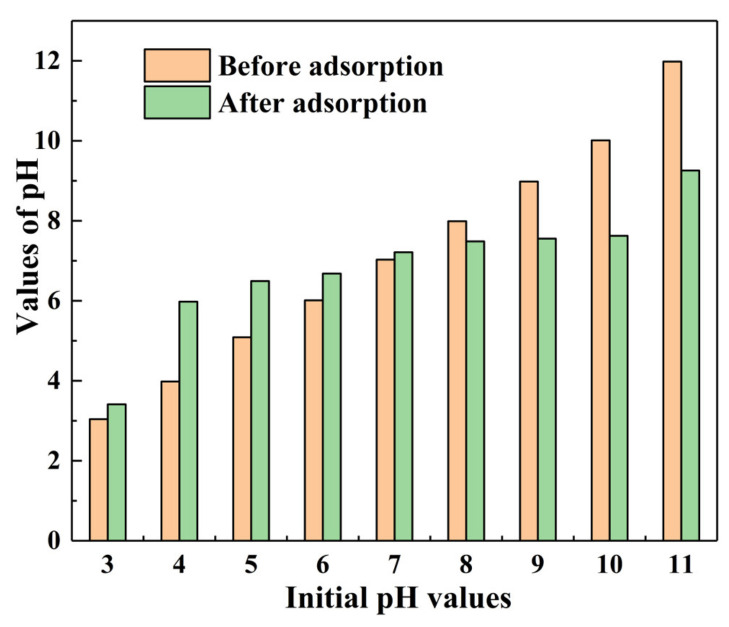
pH change of Cr(VI) adsorption equilibrium at initial pH of 3 to 11 Changes in pH during Cr(VI) removal (or adsorption) at 298.15 K.

According to XRD, FT-IR, SEM-EDS, and XPS analysis and batch experimental results, there were two main factors affecting the adsorption of Cr(VI). One was the form of iron (α–FeO(OH)) and the functional groups (–OH, C–OH, Fe–O, etc.), and the other was the pH value of the aqueous solution. Figure 14 shows the pH changes of the solution after the Cr(VI) adsorption reached equilibrium. This showed that the pH value rose in acidic conditions when adsorption equilibrium was attained. This was due to the Fe–OH on the surface of α–FeO(OH)/GOCS being protonated to Fe–OH_2_^+^. Then, Cr(VI) was adsorbed by Fe–OH_2_^+^ combination with HCrO_4_^−^ and Cr_2_O_7_^2−^ under electrostatic attraction. The pH value of the solution decreased after Cr(VI) adsorption under alkaline conditions, indicating the release of H^+^. This was mainly related to the deprotonation of Fe–OH_2_^+^. It could be seen that electrostatic attraction was one of the adsorption mechanisms for Cr(VI) removal. This result confirmed the analysis of the Fe–O peak in FT-IR and Fe–OH in XPS, and was listed as follows:

(13) $$\mathrm{Fe}-\mathrm{OH}\left(s\right)+H^{+}\left(l\right)↔\mathrm{Fe}-{\mathrm{OH}}_{2}^{+}\left(s\right)$$

(14) $$\mathrm{Fe}-2{\mathrm{OH}}_{2}^{+}\left(s\right)+{\mathrm{Cr}}_{2}O_{7}^{2-}\left(l\right)\to \mathrm{Fe}-{\mathrm{Cr}}_{2}O_{7}\left(s\right)+H_{2}O\left(l\right)$$

(15) $$\mathrm{Fe}-{\mathrm{OH}}_{2}^{+}\left(s\right)+{\mathrm{HCrO}}_{4}^{-}\left(l\right)↔\mathrm{Fe}-{\mathrm{HCrO}}_{4}\left(s\right)+H_{2}O\left(l\right)$$

However, XPS analysis results showed that there was Fe (II) and Cr(III) on the surface of the microsphere. However, Fe(II) was not involved in reducing Cr(VI). The EDS analysis results showed that the O content on the microsphere surface increased after Cr(VI) adsorption. No Cr (III) was detected in the aqueous solution after adsorption. This indicated that adsorbed Cr(VI) was reduced by the hydroxyl groups (C–OH), and then the resulting Cr(III) precipitated in the form of Cr_2_O_3_ and/or Cr(OH)_3_ on the surface of the microspheres:

(16) $$6\;\left[C-\mathrm{OH}\left(s\right)\right]+14H^{+}\left(l\right)+12e^{-}+{\mathrm{Cr}}_{2}O_{7}^{2-}\left(l\right)\to {\mathrm{Cr}}_{2}O_{3}\left(s\right)+10H_{2}O\left(l\right)$$

(17) $$3\;\left[C-\mathrm{OH}\left(s\right)\right]+10H^{+}\left(l\right)+7e^{-}+{\mathrm{HCrO}}_{4}^{-}\left(l\right)\to {\mathrm{Cr}}^{3+}\left(l\right)+7H_{2}O\left(l\right)$$

(18) $${\mathrm{Cr}}^{3+}\left(l\right)+3{\mathrm{OH}}^{-}\left(l\right)\to \mathrm{Cr}\left({\mathrm{OH}}_{3}\right)\left(s\right)$$

## 4. Conclusions

In this study, α–FeO(OH)/GOCS microspheres were prepared and used for Cr(VI) removal from aqueous solutions to examine their performance and adsorption mechanisms and to evaluate the possibility of secondary pollution. The following conclusions were summarized:

(1) The factors of pH, *m*/*v*, coexisting ions, *t*, *T,* and *C*_0_ could affect Cr(VI) adsorption on the microspheres. The optimal conditions for Cr(VI) adsorption were pH = 3, *m*/*v* = 1.0 g/L, *t* = 3840 min, *T* = 308.15 K, and *C*_0_ = 25 mg/L. In addition, the *q_e_* value decreased with the increase in pH, *m*/*v*, and coexisting ions. However, the *q_e_* value decreased with increasing *C*_0_, *t*, and *T*. The existence of SO_4_^2−^ and Ca^2+^ could significantly decline Cr(VI) adsorption, and the *q_e_* value was reduced with the rise of the molar concentration of the anion and cation. In addition, α–FeO(OH)/GOCS microspheres did not cause secondary contamination during Cr(VI) adsorption, and the removal rate remained at 97.69% at pH = 3.
(2) The adsorption of Cr(VI) was well fitted to the pseudo-second-order kinetic model with the determination coefficient (*R*^2^ = 0.9963), indicating that the process of adsorption was spontaneous and endothermic. Meanwhile, the adsorption of Cr(VI) followed the Langmuir and Sips models, suggesting heterogeneous monolayer adsorption. The maximum adsorption capacity of Cr(VI) was 63.19 mg/g at 308.15 K, which is much higher than the reported value in most similar composites.
(3) The results of the performance evaluation indicated that the α–FeO(OH)/GOCS had a good effect on Cr(VI) adsorption and did not cause secondary pollution to the environment. After being modified by α–FeO(OH), the microspheres had various functional groups, such as C–O, C = C, C–H, C–OH, –NH_2_, –COOH, and Fe–O. Functional groups of –OH, C–OH, C–H, and Fe–O were involved in binding the Cr(VI) anions to α–FeO(OH)/GOCS, and the electrostatic attraction of Fe–OH^2+^ was one of the adsorption mechanisms for Cr(VI) removal. In addition, the adsorbed Cr(VI) was reduced by the hydroxyl groups (C–OH), and then the resulting Cr(III) precipitated in the form of Cr_2_O_3_ or Cr(OH)_3_ on the surface of the microspheres.

## Figures and Tables

**Table 1:** Parameters of the adsorption kinetics model.

Adsorbed Substance	Temperature	Pseudo-First-Order		
Cr(VI)	298.15 K	*q_e_* (mg/g)	*K*_1_	*R* ^2^
		24.16	0.0020	0.9647
		**Pseudo-second-order**		
		*q_e_* (mg/g)	*K*_2_	*R* ^2^
		26.10	0.0002	0.9963

The actual pH value of the aqueous solution was 3.0, the initial concentration of Cr(VI) was 25 mg/L, and the reaction time was 3840 min. The adsorption was carried out at an oscillation rate of 170 rpm and a temperature of 298.15 K.

**Table 2:** Parameters of the Weber-Morris model.

Adsorbed Substance	Temperature	Time/min	Parameter	
Cr(VI)	298.15 K	0–180	*K_id_*_1_	*C_i_*_1_
			0.7876	–1.0253
		300–1260	*K_id_*_2_	*C_i_*_2_
			0.5503	3.8360
		1860–3840	*K_id_*_3_	*C_i_*_3_
			0.0336	22.3646

The actual pH value of an aqueous solution was 3.0, the initial concentration of Cr(VI) was 25 mg/L, and the reaction time was 3840 min. The adsorption was carried out at an oscillation rate of 170 rpm and a temperature of 298.15 K.

**Table 3:** Adsorption isothermal model parameters.

Temperature (K)	Langmuir			Freundlich			Sips			
	*q_m_* (mg/g)	*K_L_*	*R* ^2^	*K_F_*	*n*	*R* ^2^	*q_m_* (mg/g)	*K_s_*	1/*m*	*R* ^2^
298.15	56.58	0.030	0.9673	8.22	2.97	0.8337	49.37	0.006	1.61	0.9885
308.15	73.68	0.022	0.9804	7.69	2.58	0.8765	63.19	0.005	1.51	0.9964
318.15	77.62	0.022	0.9650	8.28	2.61	0.8410	64.77	0.002	1.76	0.9962

The actual pH value of the aqueous solution was 3.0, the initial concentration of Cr(VI) was 25 mg/L, and the reaction time was 3840 min. The adsorption was carried out at an oscillation rate of 170 rpm and a temperature of 298.15 K.

**Table 4:** Thermodynamic parameters.

Temperature (K)	Δ*G^θ^* (kJ/mol)	Δ*S^θ^* [J/(mol·K)]	Δ*H^θ^* (kJ/mol)
298.15	–10.28	179.75	43.31
308.15	–12.08		
318.15	–13.87		

## Data Availability

All data used during the study appear in the submitted article.

## Supplementary Materials

The following supporting information can be downloaded at: https://www.mdpi.com/article/10.3390/ma15144909/s1, Figure S1: SEM images of GOCS (a, b), α–FeO(OH)/GOCS before (c, d)/after (e, f) adsorption, Figure S2: Cr(VI) removal rate and Fe concentration change with pH value, Figure S3: The *R_L_* of Cr(VI) adsorbed by α–FeO(OH)/GOCS, Figure S4: GOCS (a) and α–FeO(OH)/GOCS (b) microspheres.
