# Supplementary material for: Removal of Cr(VI) from Wastewater Using Graphene Oxide Chitosan Microspheres Modified with α–FeO(OH)
Source: Materials (Basel). 2022 Jul 14;15(14):4909. doi: 10.3390/ma15144909 (PMC9319010; doi:10.3390/ma15144909)
Supplement: Supplementary file 1 [file materials-15-04909-s001.zip › materials-1787494-supplementary.pdf]

Supplementary Materials

Removal of Cr(VI) from Wastewater Using Graphene Oxide Chitosan Microspheres Modified with α–FeO(OH)

Yunquan Liu [1,2,3], Huimei Shan [1,2,3,*], Chunya Zeng [1,2,3], Hongbin Zhan [4,*] and Yanyue Pang [1,2,3]

1 Guangxi Key Laboratory of Environmental Pollution Control Theory and Technology, Guilin University of Technology, Guilin 541004, China; buddychampion@163.com (Y.L.); 15078381513@163.com (C.Z.); pangyanyue080512@163.com (Y.P.)
2 Collaborative Innovation Center of Water Pollution Control and Water Security in Karst Area,
Guilin University of Technology, Guilin 541004, China
3 College of Environmental Science and Engineering, Guilin University of Technology, Guilin 541004, China
4 Department of Geology and Geophysics, Texas A&M University, College Station, TX 77843, USA
* Correspondence: shanhuimei@glut.edu.cn (H.S.); zhan@tamu.edu (H.Z.); Tel.: +1-(979)-862-7961 (H.Z.);
Fax: +1-(979)-845-6162 (H.Z.)

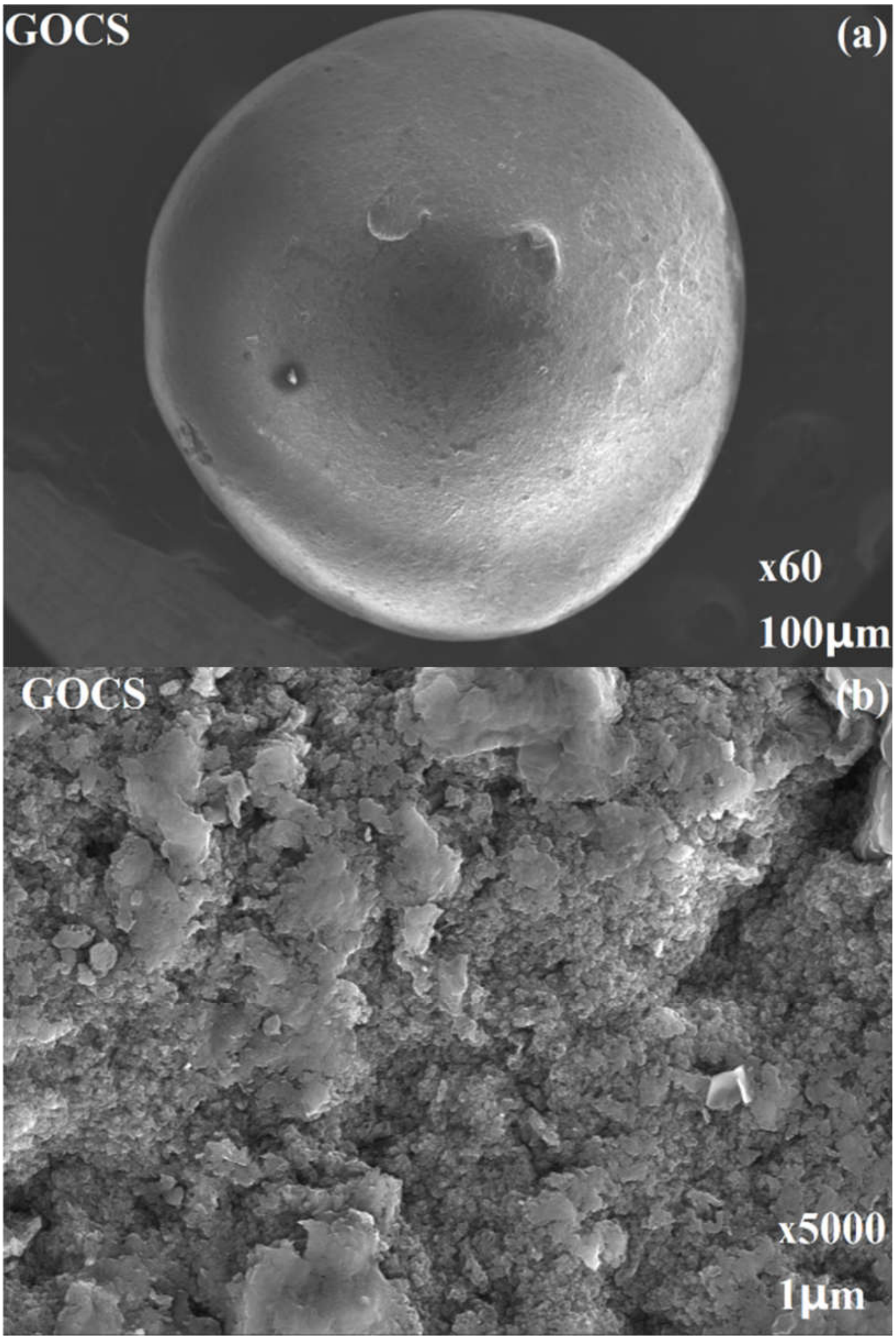
GOCS
(a)
x60
100μm
GOCS
(b)
x5000
1μm

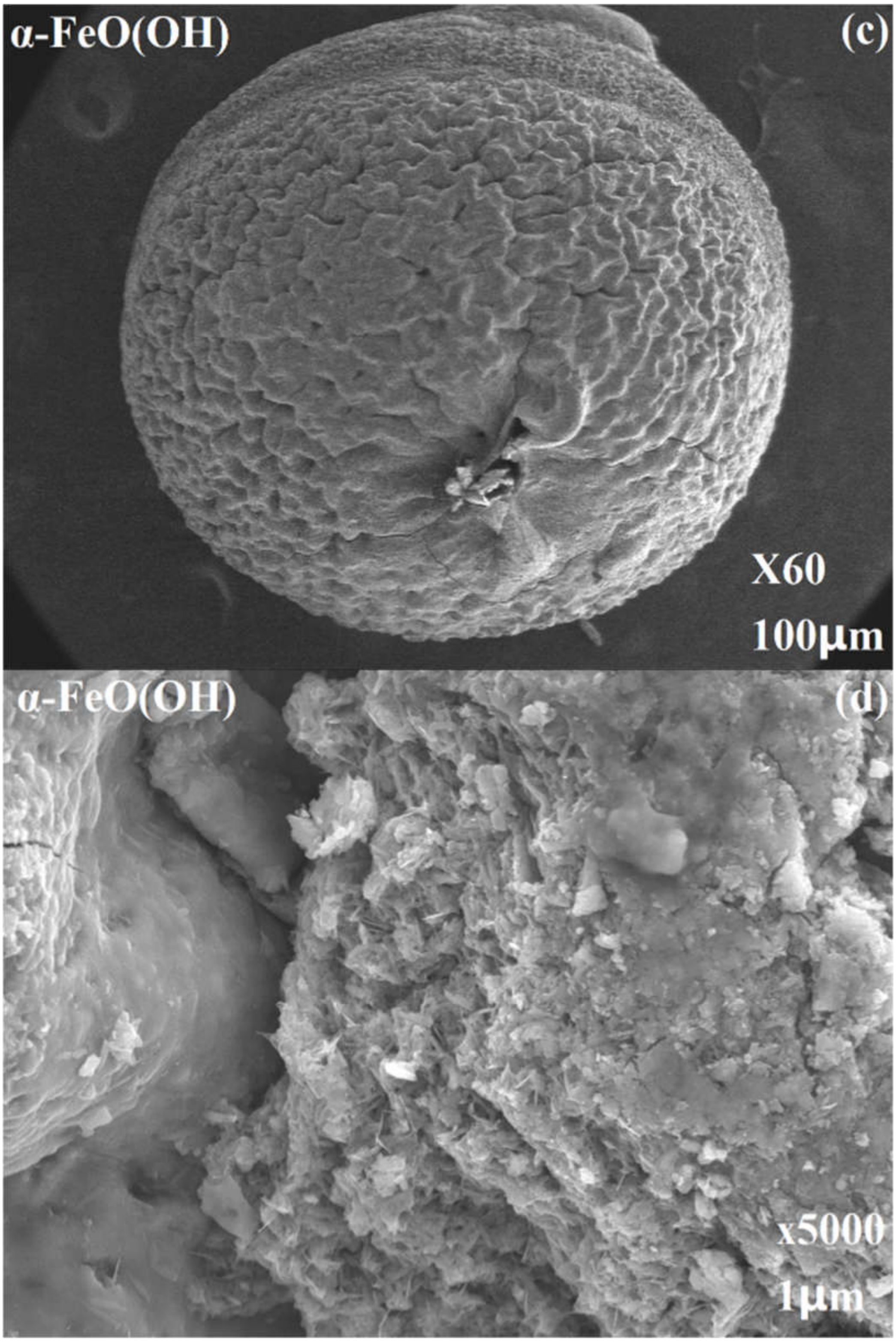
α-FeO(OH)
(c)
X60
100μm
α-FeO(OH)
(d)
x5000
1μm

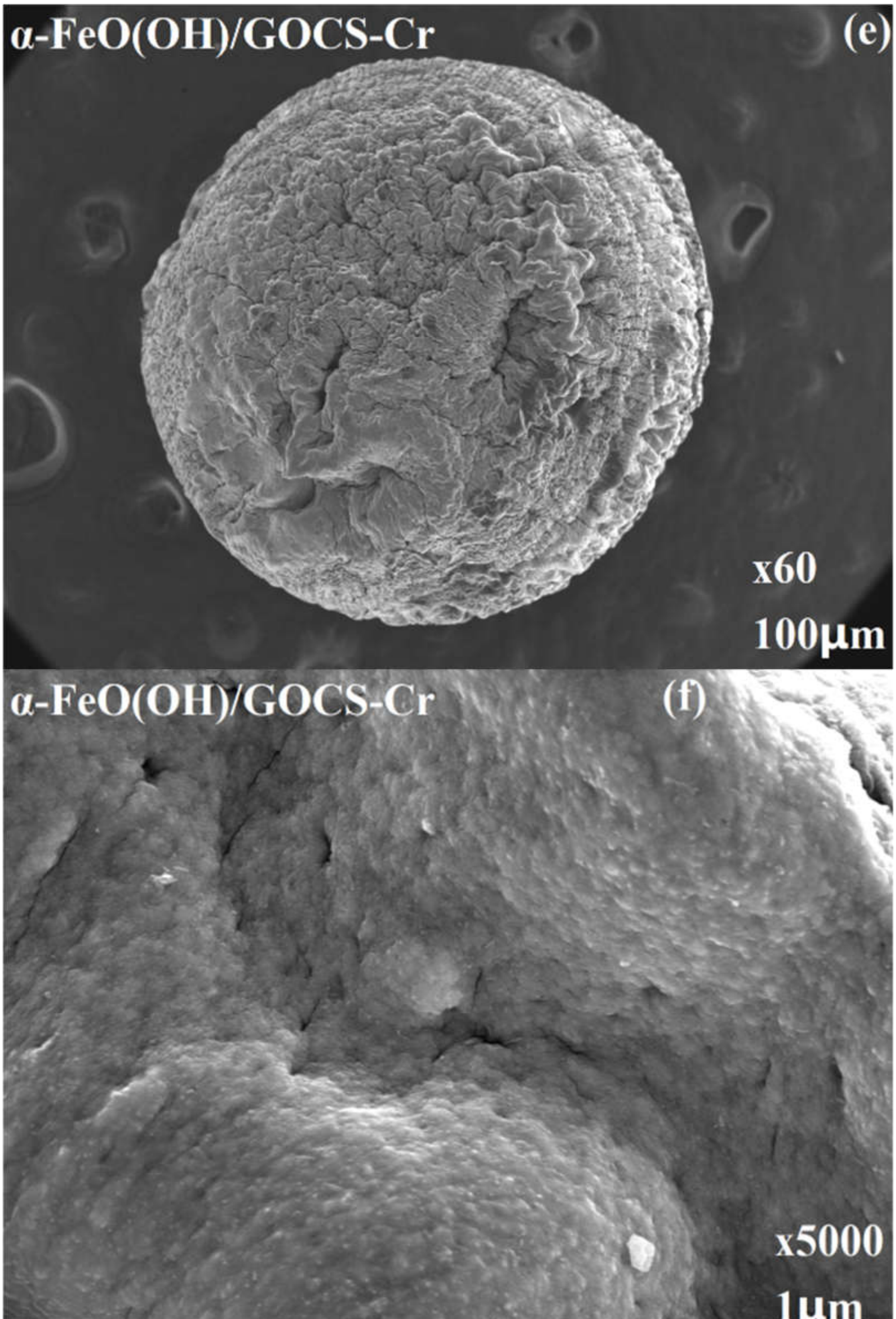

Figure S1. SEM images of GOCS(**a**,**b**), α–FeO(OH)/GOCS before (**c**,**d**)/after (**e**,**f**) adsorption.

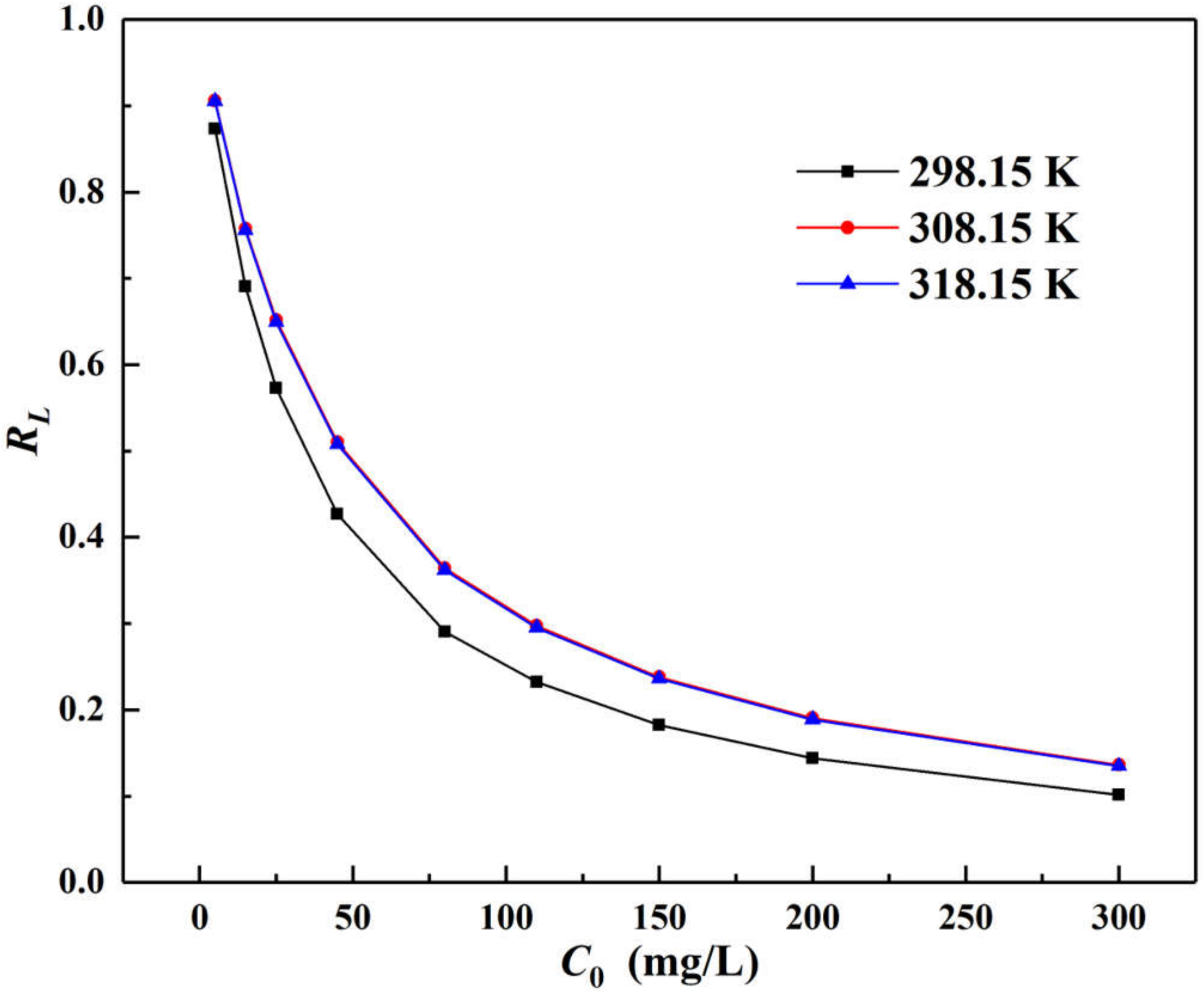

Figure S2. R_L of α–FeO(OH)/GOCS adsorbs Cr(VI).

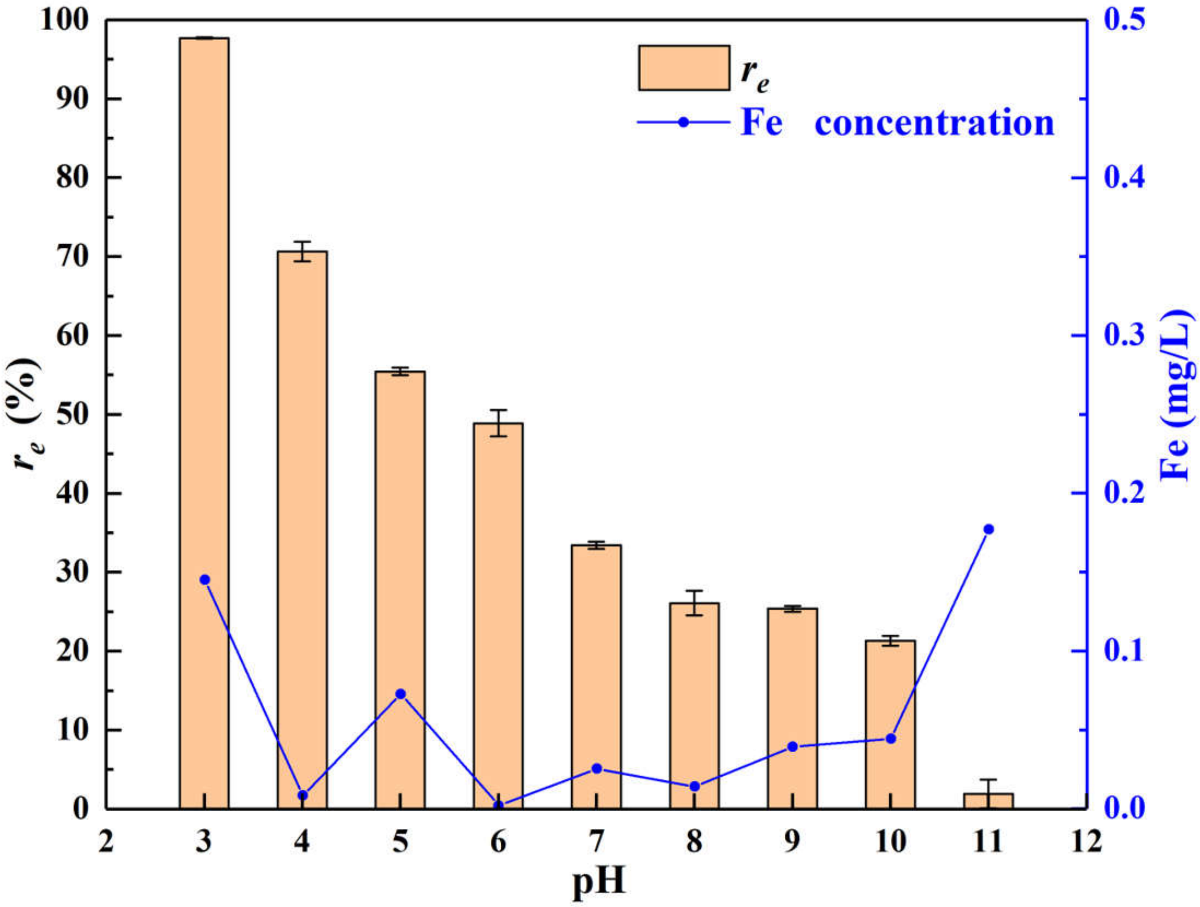

Figure S3. Cr(VI) removal rate and Fe concentration change with pH value. (The actual pH value of aqueous solution is 3.0, the initial concentration of Cr(VI) is 25 mg/L, and the reaction time is 3840 min. The adsorption is carried out at an oscillation rate of 170 rpm and a temperature of 298.15 K.).

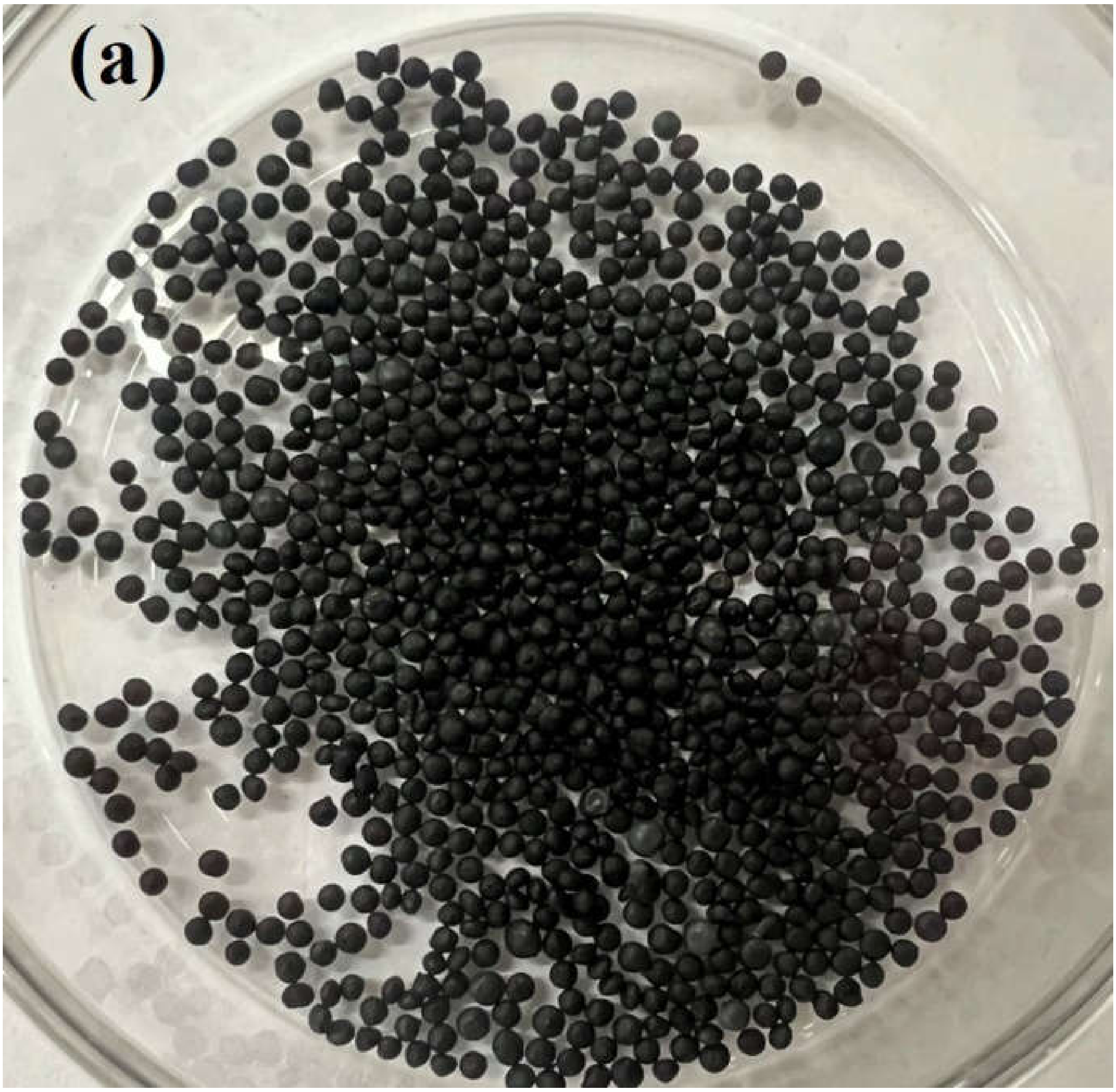
(a)

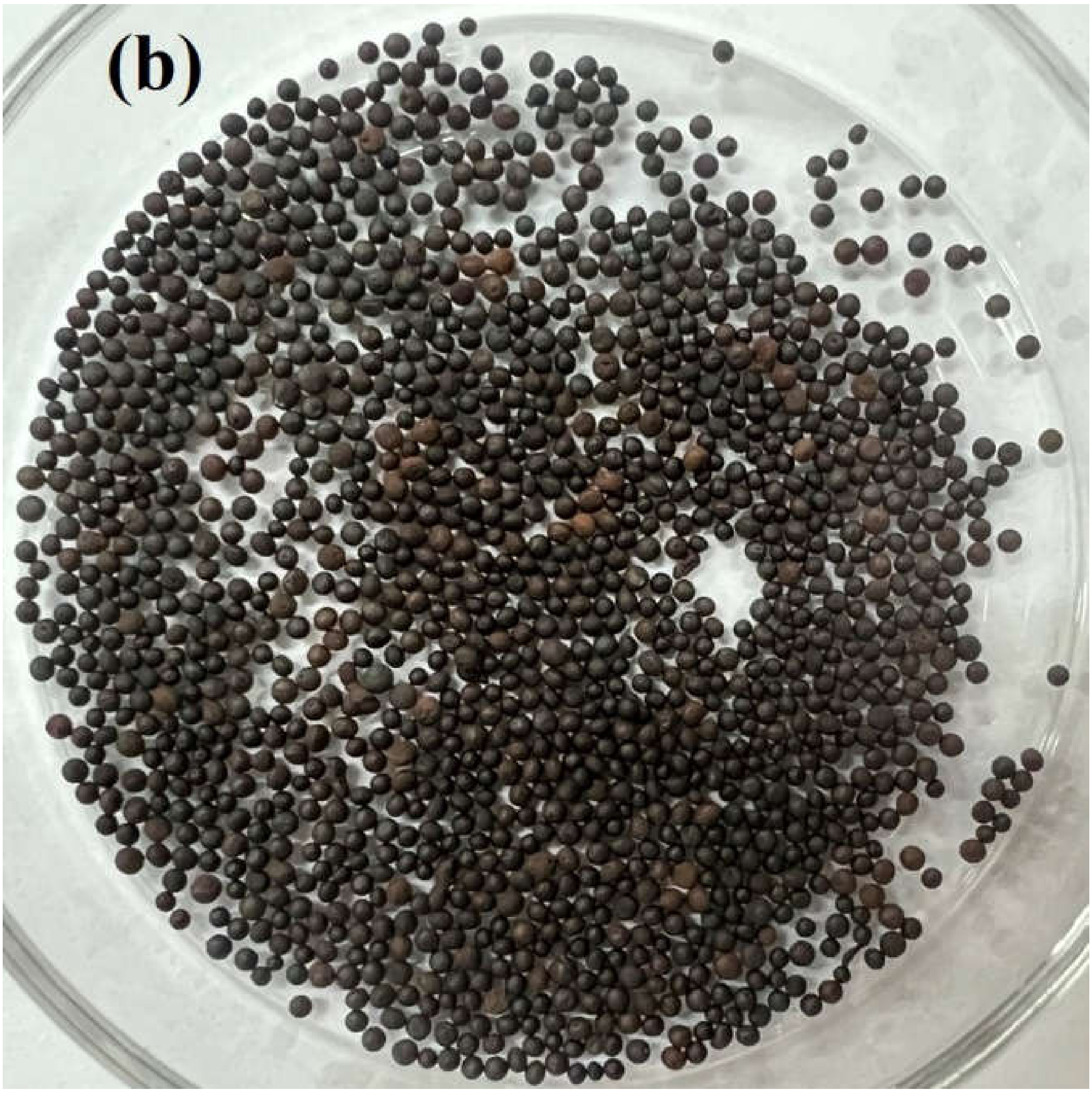

Figure S4. GOCS(**a**) and α–FeO(OH)/GOCS(**b**) microspheres.
